# Subpectoral Implantation of Cardiovascular Implantable Electronic Device: A Reasonable Alternative for the Conventional Prepectoral Approach

**DOI:** 10.29252/wjps.8.2.163

**Published:** 2019-05

**Authors:** Sung-Hwan Kim, Bommie Florence Seo, Young Choi, Ju Youn Kim, Yong-Seog Oh

**Affiliations:** 1 Division of Cardiology, Department of Internal Medicine, Seoul St. Mary’s Hospital, College of Medicine, The Catholic University of Korea, Seoul, Republic of Korea;; 2 Department of Plastic Surgery, Uijeongbu St. Mary’s Hospital, College of Medicine, The Catholic University of Korea, Seoul, Republic of Korea;; 3 Division of Cardiology, Department of Internal Medicine, Uijeongbu St. Mary’s Hospital, College of Medicine, The Catholic University of Korea, Seoul, Republic of Korea

**Keywords:** Cardiovascular, Subpectoral, Prepectoral, Implantation, Cosmetic

## Abstract

**BACKGROUND:**

The prepectoral implantation technique has been the standard procedure for cardiovascular implantable electronic device (CIED). However, it cannot be performed in such patients with thin skin or patients with cosmetic concerns. This study was designed to demonstrate the feasibility and safety of the subpectoral compared to the prepectoral approach.

**METHODS:**

We conducted a retrospective, nonrandomized comparison of the prepectoral (234 cases) and subpectoral approach (32 cases) in patients who received CIED implantation at a tertiary center between July 2012 and May 2015. We compared lead characteristics, procedure time and complications between the subpectoral and prepectoral approach.

**RESULTS:**

In the subpectoral group, two complications were observed, whereas six complications were found in the prepectoral group (2/32 vs. 6/234, respectively, *p*=0.25). In the subpectoral group, one patient developed wound infection and the others were safely conducted without any complications. In the prepectoral group, two patients developed hemopericardium, three developed pocket hematoma requiring surgical revision, and one developed a pneumothorax. Procedure time in the subpectoral group took longer than that in the prepectoral group (150±50 min versus 91±49 min, *p*=0.06). In lead characteristics, there were no significant differences between the two groups.

**CONCLUSION:**

The subpectoral approach is technically feasible and non-inferior to the prepectoral approach, in the aspect of complication and lead characteristics, but seemed to take more procedure time. The subpectoral approach is a more reasonable choice for selected patients in whom the prepectoral approach is not feasible or in individuals who have cosmetic concerns.

## INTRODUCTION

The standard prepectoral approach for cardiovascular implantable electronic device (CIED) results in placement of the pulse generator in the subcutaneous tissue of the upper chest, superficial to the pectoralis major muscle. It has been widely used by cardiologists, because the subcutaneous space is conveniently accessible. While the prepectoral approach is well tolerable in most patients, it may not be appropriate for patients who have thin skin or indwelling catheters. Even, if prepectoral implantation has been done safely without any immediate complications, it is prone to erosion through overlying skin, and results in an unattractive bulge in the anterior aspect of the chest, causing cosmetic problems especially in young women or children. As a solution, some studies have reported infrapectoral approach as an alternative consideration.^1^^,^^2^

However, infrapectoral approach involves dissection and elevation of the pectoralis major muscle, which is a more complicated procedure with an increased risk of bleeding. In 1995, Foster *et al.* introduced six successful implantations in the subpectoral position using a subclavian incision and a vertical axillary incision.^3^ Subpectoral approach without transaction of the pectoralis major muscle has been reported.^4^^,^^5^ However, all previous reports focused on the aesthetic perspective and did not address CIED function which is the ultimate goal of the implantation. Previous reports were too small to investigate adverse events and had no control group which could be compared with.^4^^,^^5^

The recently downsized devices were not included in the previous studies. The purpose of this study was to compare subpectoral and conventional prepectoral approach for CIED implantation in terms of procedure related factors (procedure time, lead characteristics and complication) and patient related factors (pain, cosmetic satisfaction) and to show the subpectoral approach is not inferior to the prepectoral approach, and may even be a better choice for some patients. 

## MATERIALS AND METHODS

We conducted a retrospective, nonrandomized comparison of the subpectoral and prepectoral approaches in patients who received pacemaker or implantable cardioverter-defibrillator (ICD) implantation between July 2012 and May 2015 at Seoul St. Mary’s Hospital, Republic of Korea. All consecutive patients who underwent new implantation of a permanent pacemaker or ICD were enrolled. Revisional cases of patients with preexisting pacemakers or ICDs who received a pocket change from prepectoral to subpectoral were also included. Patients who underwent cardiac resynchronization therapy were excluded. 

The choice of implantation method had left to the discretion of the operator. All patients provided written informed consent. In cases of the subpectoral approach, the plastic surgery department performed approach and formation of the pocket. The prepectoral approach was done in the conventional method by the cardiologist. All procedures were performed in the electrophysiology laboratory under intravenous anesthesia, and lead insertion was done by the cardiologist. Patients’ characteristics including age, sex, medical problems and reason for CIED insertion were reviewed. We compared lead characteristics, procedure time and complications between the subpectoral and prepectoral approaches.

The conventional prepectoral implantation was performed by the cardiology team. A horizontal line of 5~6 centimeters was designed in the left subclavicular area inferior to the level of the axillary vein. The skin incision was followed by subcutaneous adipose tissue layer dissection. The leads were inserted into the axillary vein via this opening, and then the adipose tissue was further dissected to provide sufficient space for the device. After the device was inserted and the leads were connected, the subcutaneous tissue was closed with absorbable sutures and the skin was approximated using metallic skin staples. 

**Fig. 1:**
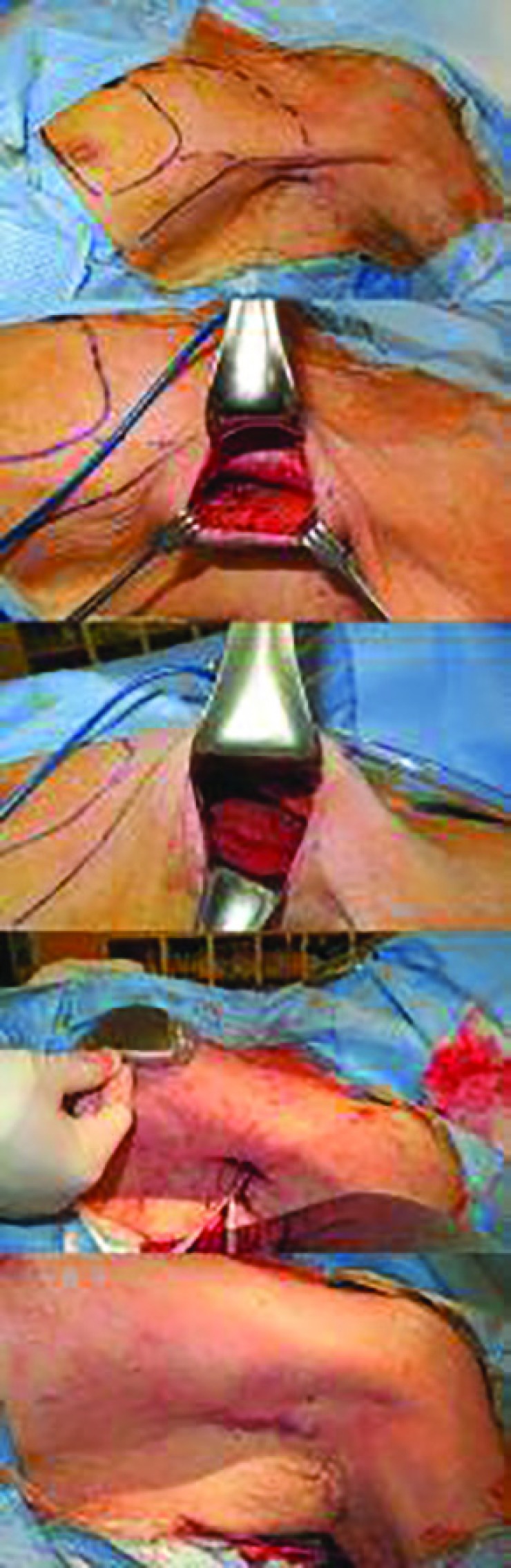
**(A)** The axillary incision is seen behind the anterior axillary line. The extent of the subpectoral pocket is drawn in broken lines, and the predicted location of the device is also drawn in the vicinity of the nipple. **(B)** After skin incision and dissection to the lateral border of the pectoralis major muscle. **(C)** The pectoralis major muscle has been pulled upward and the subpectoral space is easily visualized. **(D)** The cardiologist uses this approach to insert leads, which are seen extending out of the skin incision. The surgeon estimates the location of the device and checks if the leads will reach the device without tension. **(E) **After the device is connected to the leads and inserted in the planned subpectoral plane, the skin is closed using absorbable sutures

For De novo implantation, the plastic surgery team designed the incision around 2 centimeters posterior to the anterior axillary line with the patient in a sitting or standing position to ensure the scar was not visible from anterior view. The incision was 4 centimeters at maximum, with its cranial border around 1 centimeter from the axillary apex (Figure 1). The left shoulder was abducted 90 degrees, and the neck was rotated in the opposite direction to maximize procedural space. A solution of 2% lidocaine, 1:100,000 epinephrine and saline was injected along the predesigned line. 

The skin incision was made and dissection through the subcutaneous adipose tissue was performed toward the lateral margin of the pectoralis major muscle. After identifying the pectoralis major, the subpectoral space was recognized and dissected, taking care to provide meticulous bleeding control. The extent of the pocket was cranially until the clavicle could be palpated, caudally just below the level of the nipple, and medially to the extent that the device could fit comfortably in the pocket without risk of lateralization.

The cardiology team then inserted the leads into the axillary vein. During the first year (July 2012 to June 2013), a separate horizontal incision was made at the level of insertion in the infraclavicular skin. The horizontal incision was at maximum 4 cm. The right ventricle (RV) pacing lead was inserted through the puncture site and placed in the RV apex or septum. The right atrium (RA) pacing lead was inserted and placed in the RA appendage. The device was connected to the leads, and after confirmation that the leads were positioned well, the RV and RA pacing leads were tested and affixed. 

The device was then positioned in the subpectoral pocket, at the level of the nipple or slightly more cranially. Anchoring of the device was done with an absorbable suture. The lateral portion of the pectoralis major fascia was approximated to the serratus anterior fascia with a #3-0 Vicryl suture to prevent lateral extrusion. Subcuticular sutures were performed with #4-0 polydioxanone sutures, and the skin was approximated with surgical taping. No drain was placed into the pocket, and light compression was provided with foam dressings.

For revisional implantation in patients with preexisting devices that required change of device pocket, the same incision and approach was conducted to secure the subpectoral space. The previous device was disconnected from the leads and removed either through the same axillary incision or through a separate skin incision. The previous prepectoral space was inspected, and debrided of any necrotic or suspicious tissue. The leads were then pulled through the pectoralis major muscle down to the subpectoral space after blunt dissection using Mosquito forceps. 

The device to be implanted was then seated in the subpectoral space as described above, and the leads were connected to the CIED in the same method. Closure and dressings were done in an identical manner. A complication was defined as an event that required surgical or medical intervention. This included pneumothorax, hemothorax, cardiac tamponade, pocket hematoma requiring surgical revision, pocket infection requiring device removal and any unexpected revision.

For statistical analysis, continuous variables were expressed as mean±SD. Parametric data were expressed as mean±standard deviation and nonparametric data as median (interquartile range). Data was compared using Students’ paired *t*-test. Categorical variables, expressed as numbers and percentages, were compared using Chi-square or Fisher’s exact test. The cumulative complication free curves were generated by Kaplan-Meier method and comparisons between two curves were made using the log-rank statistics. All tests of significance were two-tailed, and *p*<0.05 was considered significant.

## RESULTS

**Fig. 2:**
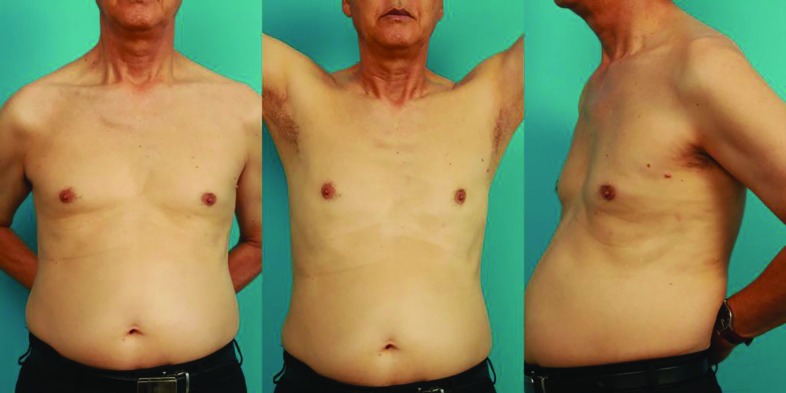
**(A) **72 year-old male who underwent subpectoral ICD insertion two months ago. **(B, C)** The short axillary incision scar is visible only when the patient’s arms are held up, and even then only barely

**Fig. 3:**
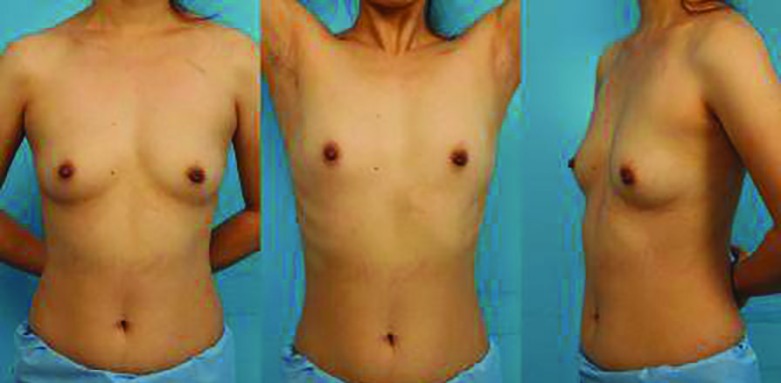
A 35 year-old female patient who underwent a subpectoral implantation 11 months ago with an additional subclavian venous approach. **(A) **The subclavian scar is visible upon anterior view. There is no asymmetry of the breasts. **(B, C)** The axillary incision is barely discernible even when the patient lifts her arms up

Of the 266 patients who underwent CIED implantation at Seoul St. Mary’s Hospital, the subpectoral implantation was performed in 32 (12%) patients, whereas 234 (88%) patients underwent conventional prepectoral implantation. Of the 32 patients who underwent subpectoral approach, new implantations of CIED were in 24 of 32 patients (Figure 2) and 8 of 32 patients received a change from a previous prepectoral pocket to the subpectoral pocket (Figure 3). The clinical characteristics of both groups were shown in Table 1 and the CIED characteristics in Table 2. 

**Table 1:** Baseline characteristics of patients undergoing cardiovascular implantable electronic device implantation

**Variable**	**Prepectoral** **(n=234)**	**Subpectoral** **(n=32)**	***p*** ** value**
Age (year)	67±14	47±20	<0.01
Male (%)	111 (47)	16 (50)	0.65
Diabetes Mellitus (%)	25 (11)	0 (0)	0.04
Hypertension (%)	64 (27)	2 (6.3)	0.08
Previous stroke (%)	9 (3.8)	3 (9.4)	0.18
Chronic kidney disease (%)	12 (5.1)	1 (3.1)	0.60
Coronary artery disease	16 (6.8)	2 (6.3)	0.68
Atrioventricular block (%)	78 (33)	6 (19)	0.14
Sick sinus syndrome (%)	117 (50)	5 (16)	<0.01

**Table 2:** Baseline characteristics of implanted devices

**Variable**	**Prepectoral** **(n=234)**	**Subpectoral** **(n=32)**	***p*** ** value**
Pacemaker mode			
DDD (%)	195 (83)	20 (63)	<0.01
VVI (%)	36 (15)	11 (34)	<0.01
VDD (%)	2 (0.9)	0 (0)	0.60
AAI (%)	1 (0.4)	1 (3.1)	0.09
Puncture site			
Subclavian vein (%)	56 (24)	12 (38)	0.15
Axillary vein (%)	178 (76)	20 (63)	0.15
Device Type			
Pacemaker (%)	195 (83)	15 (47)	<0.01
Implantable Cardioverter-Defibrillator (%)	39 (17)	17 (53)	<0.01

DDD: Dual chamber pacemaker; VVI: Single-lead ventricular pacemaker; AAI: Single-lead atrial pacemaker; VDD: Atrial-floating single ventricular lead

**Fig. 4:**
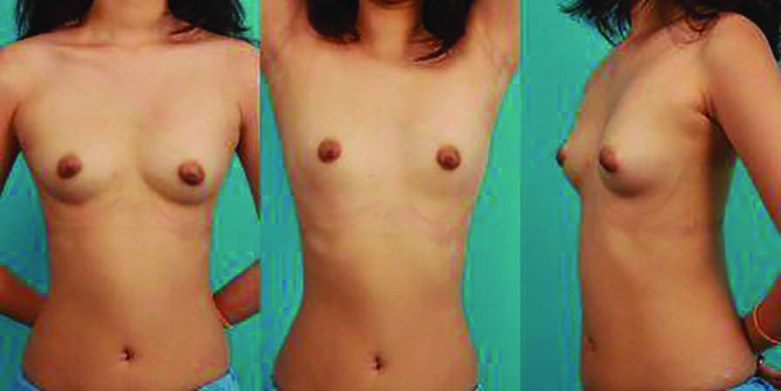
A 23 year-old female patient who underwent subpectoral implantation four months ago via the axillary approach**. (A, B, C) **The patient’s breasts are symmetric and the axillary scar is barely visible

The patients in the subpectoral approach group were significantly younger than those in prepectoral group (47±20 versus 67±14 years, *p*<0.01). Sex distribution was similar (male, 50% in the subpectoral group versus 47% in the prepectoral group, *p*=0.65). In patients with the subpectoral approach, surgical scar was invisible from anterior view due to its hidden location behind the anterior axillary line, but was almost indiscernible even when visualized. The device and pocket was undetectable to the eye because of its placement behind supple soft tissue (Figure 4). 

The reasons that the subpectoral approach was chosen were cosmetic concerns (n=29), CIED infection of the previous prepectoral pocket (n=2), and discomfort of previous prepectoral pocket (n=1). The two patients who underwent this pocket change due to previous prepectoral pocket infection presented with wound site erosion (n=1) and purulent infection (n=1). The median time between removal of device and reimplantation was 11 days. Lead extractions were attempted in these two patients but were unsuccessful. The ages of the leads were 63 and 70 months. Both were bipolar active fixation leads. 

All CIED (15 pacemakers, 17 ICD) were successfully reimplanted in the subpectoral position as described earlier. A total of two patients underwent reimplantation after the initial subpectoral approach. The reasons for reimplantation were device pocket infection and intractable pain after previous procedure. Subpectoral implantation required longer procedure duration (150±50 min versus 91±49 min, *p*=0.06), but was statistically insignificant. Follow up duration in the prepectoral group was significantly longer than that in the subpectoral group (659±365 days versus 607±558 days, *p*<0.01).

The complication rates differed (2/32 (6.3%) in the subpectoral group versus 6/234 (2.6%) in the prepectoral group *p*=0.25). Of the prepectoral group, postoperative bleeding occurred in five cases (three cases of pocket bleeding, two cases of hemopericardium) and pneumothorax was found in one case during the procedure but all patients except one patient who underwent device extraction due to unsolved wound infection were successfully and safely recovered without any sequelae. 

In the subpectoral group, 26/32 (81.3%) of patients were very satisfied with the procedure cosmetically and did not complain of any discomfort, but 6/32 (18.7%) were not satisfied with the procedure. The reasons for dissatisfaction were skin abrasion (n=1, 17%), which completely healed with seven days of oral antibiotics and wound dressing, pain or discomfort (n=3, 50%), which disappeared spontaneously within two months. But two patients underwent reimplantation. One patient underwent reimplantation nine months after first procedure due to intractable pain with no response to any pain medications. 

**Fig. 5:**
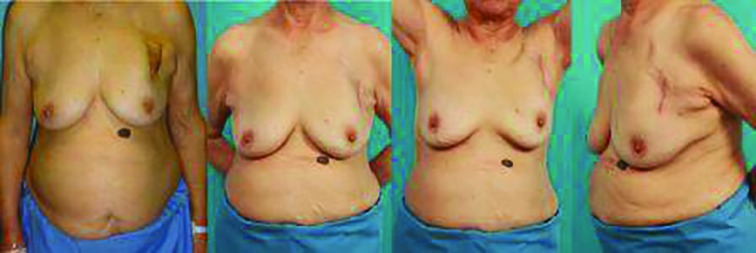
A 79 year-old female patient who underwent conventional prepectoral implantation. **(A)** The skin overlying the device had become very thin and showed infection signs. **(B, C, D)** After pocket change to the subpectoral pocket and excision of necrotic skin. the patient no longer could palpate the device, and risk of device protrusion was minimized

One patient who received subpectoral approach due to CIED pocket infection underwent device and lead removal because of a persistent infection in the axillary incision site despite serial wound debridement and antibiotic therapy for seven months. At the beginning of subpectoral implantation of CIED, lead redundancy was observed to diminish in post-implantation X-ray, though lead function was acceptable (Table 3, Figure 5). To prevent lead dislodgement, we made sure to lead tagging to pectoralis minor muscle and gave more redundancy of lead during insertion. 

**Table 3:** Procedure outcomes of the leads

**Variable**	**Prepectoral** **(n=234)**	**Subpectoral** **(n=32)**	***p*** ** value**
Atrial lead			
P wave amplitude (mV)	2.5±1.3	2.6±1.3	0.90
Pacing thresholds (V at 0.5 ms)	0.8±0.3	0.9±0.6	0.08
Impedance (Ω)	498.2±118.4	489.3±88.7	0.72
Ventricular lead			
R wave amplitude (mV)	9.7±4.4	10.6±5.0	0.31
Pacing thresholds (V at 0.5 ms)	0.8±1.0	0.8±0.6	0.98
Impedance (Ω)	652.4±212.5	497.8±141.4	<0.01
Defibrillation			
Thresholds (V)	16.9±8.8	22.5±17.7	0.45
High impedance (Ω)	55.8±15.1	59.8±4.9	0.56

In leads performance, there were no significant difference between two groups in terms of sensing and pacing thresholds of atrial and ventricular lead. However, impedance of ventricular lead was lower within normal limit in patients with subpectoral group. Defibrillation functions were normal and no difference between both groups. Overall, lead performance was normal in patients with subpectoral groups. 

## DISCUSSION

The present study contributes to the existing literature or knowledge with the following. First, this is the first and largest study of the subpectoral approach that is compared with the conventional prepectoral approach. Second, our subpectoral approach requires only one incisional line in the axillary area (Figure 4). Most previous studies reported their subpectoral approach required either an additional incision in the subclavicular area for vein access or in the inframammary area for generator insertion.^5^^,^^6^

Third, functional status of the leads was provided in the present study. Fourth, application was successful in most revisional cases (Figure 3), implying that this procedure may be performed in patients with preexisting prepectoral pockets that medically require or electively choose to receive this procedure. The use of CIED has sharply increased because recent technical advances have enhanced functional aspects and expanded treatment indications.^7^^-^^9^ The conventional prepectoral implantation technique has been widely used because it is easily and quickly performed without the need of surgical collaboration requiring less demanding surgical skills.^10^

However, some patients who need CIEDs are unsuitable for device implantation in the prepectoral area and require an alternative site. In such patients, there are several great advantages in the subpectoral implantation technique over prepectoral implantation. Evidently, the subpectoral approach provides supple, healthy soft tissue coverage of the device, therefore preventing skin ulcerations or erosions even in thin patients (Figure 2A).^10^ This is especially an advantage for the fragile, less elastic, thin and vulnerable cutaneous and subcutaneous tissue of our aging population, as more geriatric patients are indicated for CIED implantation.^11^

Second, in the prepectoral approach, the device was inevitably visible, causing a considerable aesthetic discomfort, especially in the young population regardless of gender. The subpectoral pocket allows the pulse generator to be hidden behind a healthy layer of muscle, which makes it almost undetectable on sight, and less palpable, rendering it in a more stable location that prevents generator migration or torsion.^12^ It is better concealed and the patient is less aware of the lateral edge of the CIED. Third, the subclavian scar in the prepectoral approach was easily visible. The subpectoral approach using the axillary incision enables both the plastic surgeon and the cardiologist an entrance to the generator pocket and the lead insertion sites, eliminating the need for a subclavian scar. 

The subpectoral approach can be applied regardless of age and skin condition. We do acknowledge that there were some limitations in the present study. First, this comparative study a retrospective and nonrandomized study conducted and performed at a single center. Second, the follow up periods of subpectoral implantation were relatively short to evaluate long-term outcome. In conclusion, we demonstrated that subpectoral approach was not inferior to prepectoral approach regarding to feasibility, safety, and function of the CIED. In addition, patients’ satisfaction rate was even higher in the subpectoral approach group. 
